# Editorial: Advances in early detection, pathophysiology, and management of mild cognitive impairment

**DOI:** 10.3389/fmed.2026.2007251

**Published:** 2026-10-12

**Authors:** Edgar Vásquez-Carrasco, Cristian Sandoval-Vásquez, Pablo Valdés-Badilla

**Affiliations:** 1 School of Occupational Therapy, Faculty of Psychology, Universidad de Talca, Talca, Chile; 2 Centro de Investigación en Ciencias Cognitivas, Facultad de Psicología, Universidad de Talca, Talca, Chile; 3 VITALIS Longevity Center, Universidad de Talca, Talca, Chile; 4 Departamento de Medicina Interna, Facultad de Medicina, Universidad de La Frontera, Temuco, Chile; 5 Department of Physical Activity Sciences, Faculty of Education Sciences, Universidad Católica del Maule, Talca, Chile; 6 Sports Coach Career, Faculty of Life Sciences, Universidad Viña del Mar, Viña del Mar, Chile

**Keywords:** biomarkers, cognitive assessment, disease management, healthy aging, mild cognitive impairment, neurodegeneration, risk factors

## Introduction

Mild cognitive impairment (MCI) is an important clinical entity that sits between the expected cognitive changes of later life and dementia, but it is far from being a uniform condition. With advances in biomarker research, neuroimaging, risk stratification, and disease-modifying treatments, the emphasis is increasingly on identifying cognitive change at an earlier stage. However, early recognition is only helpful if it is interpreted with care. Who is being assessed, what process might underpin the impairment, what is the prognosis, and what can realistically be offered in response?

These questions are addressed from different but complementary angles in the articles collected in this Research Topic, Advances in Early Detection, Pathophysiology, and Management of Mild Cognitive Impairment. The contributions span clinical screening, neurological and metabolic comorbidity, vascular and behavioral factors, biomarkers, health system pathways and non-pharmacological care. In sum, they make an important point: One test score or one biological marker cannot do full justice to MCI. Its expression is the result of the interplay of neurological, vascular, behavioral, metabolic, psychological and social factors.

Some contributions address the first step of this process, i.e., the accurate recognition of MCI. Jerjes and Harding question the use of universal cognitive screening cut-offs, given the demographic, educational, cultural and clinical differences that may affect performance. Their concept of “threshold governance” is particularly relevant today, as a threshold for screening can determine who gets specialist assessment, biomarker testing or treatment. A complementary clinical perspective on hospitalized older adults with Parkinson's disease is provided by Gong et al. MCI was common in this population and related to educational level, motor severity, anxiety, hemoglobin concentration and length of hospitalization. These studies illustrate why screening results need to be interpreted within their clinical and sociodemographic context.

This context is even more crucial in the era of disease-modifying therapies for Alzheimer's disease (AD). Kuroda et al. assessed neurodegeneration imaging biomarkers in MCI patients who may be potential candidates for lecanemab and found that MRI and SPECT markers were not reliable predictors of cerebrospinal fluid evidence of AD pathology, cautioning against using evidence of neurodegeneration as a surrogate for molecular confirmation of the underlying disease process. A practical perspective on diagnosis is provided by a real-world study in Spain by Sánchez-Juan et al., which reveals delays in the diagnostic pathway and low uptake of AD-specific biomarker testing. Together these results suggest the need for improved diagnostic tools to be complemented by functional referral pathways and access to appropriate confirmatory testing.

Other studies have broadened the concept of risk beyond traditional cognitive tests. Motoric cognitive risk syndrome was associated with incident MCI and cognitive decline in community-dwelling older adults, suggesting that a simple motor measure such as gait speed may have potential value when combined with subjective cognitive concerns (Zhang et al.). Another aspect of physiology studied by Sepúlveda-Figueroa et al. was the cardiac autonomic responses to exercise. The link between cognitive performance and dynamic heart-rate-variability trajectories supports the idea that functional challenges can expose physiological differences that are less evident at rest.

The biological heterogeneity of cognitive impairment is evident throughout the Issue. Duan et al. studied the relationship of white matter hyperintensities with mild behavioral impairment and cognition in cerebral small vessel disease. Their findings indicate that behavioral symptoms might alter the relationship between vascular brain damage and cognitive function and provide evidence for greater focus on behavioral changes when evaluating cognitive vulnerability in later life. Sun et al. examined MCI from a different analytical perspective. The authors combined community phenotyping, transcriptomic analysis, pathway enrichment and molecular docking to explore associations between MCI and AD. The combination of population and molecular data exemplifies the promise and complexity of moving from association to mechanistic hypotheses.

Another layer is neuroinflammation. In their systematic review and meta-analysis, Yang et al. reported increased levels of YKL-40 in cerebrospinal fluid and peripheral blood in the early and established phases of the AD continuum. However, YKL-40 did not clearly discriminate between MCI and AD, limiting its utility as a stand-alone staging marker. The distinction matters: a biomarker may reflect a biologically relevant process but may not provide the needed diagnostic discrimination for a particular clinical decision.

Studies of risk factors require similar methodological rigor. Wu discusses and reanalyzes evidence on MCI in type 2 diabetes, showing how the choice of statistical model can influence conclusions about potential risk factors. Beyond the specific issue addressed, this contribution highlights the importance of methodological transparency, appropriate handling of heterogeneity, and reproducibility in evidence synthesis, particularly when meta-analytic findings may ultimately inform risk stratification or clinical recommendations.

Detection, however, is only one part of the challenge. Kong et al. focus on the management of MCI in the community and the relationship between family support, social isolation and healthcare professionals and community structures. Central to their suggested Social Worker–Physician–Family framework is care coordination. Meanwhile, Jiao et al. summarize randomized evidence on visual art therapy. They found evidence of improvement in global cognition and potential benefits for memory, depressive symptoms and activities of daily living, although larger, methodologically robust trials remain necessary.

Together, this Research Topic suggests a more integrated approach to MCI. Screening thresholds are not to be applied mechanically but should be interpreted. Cognitive assessment can be augmented by measures of behavior, motor function, autonomic physiology, imaging, and molecular biomarkers, but each measure should be utilized with a specific objective. Meanwhile, improvements in diagnostic accuracy will have limited clinical utility if patients cannot efficiently navigate referral and biomarker pathways or access appropriate support after diagnosis.

To summarize, it is unlikely that the next phase of MCI research will be characterized by a single test or intervention. A more realistic goal is to develop frameworks that integrate complementary sources of information while maintaining the clinical context and acknowledging uncertainty. The studies included in this Research Topic contribute to the field in this direction. They also highlight persistent gaps such as longitudinal validation, replication across populations and healthcare settings, greater standardization of outcomes and stronger translation of research findings into routine care.

